# Acute Medical Events in Adults with Profound Autism: A Review and Illustrative Case Series

**DOI:** 10.3390/brainsci15070740

**Published:** 2025-07-10

**Authors:** Heli Patel, Anamika L. Shrimali, Christopher J. McDougle, Hannah M. Carroll

**Affiliations:** 1 T.H. Chan School of Medicine, University of Massachusetts Chan Medical School, 55 North Lake Avenue, Worcester, MA 01655, USA; heli.patel12@umassmed.edu (H.P.); hannah.carroll@umassmed.edu (H.M.C.); 2 Tufts School of Medicine, Tufts University, 145 Harrison Avenue, Boston, MA 02111, USA; anamika.shrimali@tufts.edu; 3 Lurie Center for Autism, 1 Maguire Road, Lexington, MA 02421, USA; 4 Massachusetts General Hospital, 55 Fruit Street, Boston, MA 02114, USA; 5 Department of Psychiatry, Harvard Medical School, 25 Shattuck Street, Boston, MA 02115, USA

**Keywords:** profound autism, acute medical events, intellectual disability, emergency care

## Abstract

*Background*: Autism spectrum disorder (ASD) is associated with social-communication challenges that can hinder timely diagnosis and treatment during acute medical events (AMEs). The purpose of this report is to review the literature on medical comorbidities and AMEs in adults with profound ASD and highlight how healthcare teams can better understand atypical presentations of acute pain and discomfort in adults with profound ASD to reduce delayed diagnoses, delays in treatment, and ultimately improve health outcomes. *Methods*: The literature on medical comorbidities and AMEs in adults with profound ASD was reviewed using the following databases: PubMed, PsycINFO, and Google Scholar. The histories of three adults with profound ASD who experienced AMEs—specifically, appendicitis, nephrolithiasis, and eosinophilic esophagitis (EoE)—are described. The clinical cases were selected to illustrate the challenges inherent in diagnosing and treating AMEs in adults with profound ASD in the context of the review. *Results*: In Case 1, a 31-year-old male with autism was diagnosed with perforated appendicitis after his family noticed behavioral changes. In Case 2, a 36-year-old male with autism experienced intermittent pain from nephrolithiasis and communicated his discomfort through irritability and pointing. In Case 3, a 34-year-old male with autism exhibited atypical behavior due to pain from undiagnosed EoE, identified after years of untreated pain and multiple unsuccessful clinical procedures. *Conclusions*: This review and the illustrative cases demonstrate the significant role that communication barriers play in delayed medical diagnoses for adults with profound ASD during AMEs. Integrating caregiver insights and recognizing atypical pain expressions are essential for improving the accuracy and timeliness of diagnosis and treatment in this population.

## 1. Introduction

### 1.1. Overview

Autism spectrum disorder (ASD) is a lifelong neurodevelopmental condition characterized by persistent deficits in social communication and social interaction across multiple contexts as well as restricted, repetitive patterns of behavior, interests, or activities, as defined by the Diagnostic and Statistical Manual of Mental Disorders, fifth edition (DSM-5) [1]. Intellectual disability (ID), which commonly co-occurs with ASD, is defined in the DSM-5 as having an onset during the developmental period that includes both intellectual and adaptive functioning deficits in conceptual, social, and practical domains. It is further classified into mild, moderate, severe, and profound subtypes based on the level of support required and the degree of impairment in adaptive functioning [1]. The prevalence of ASD continues to increase, affecting approximately 1 in 36 children in the United States [2]. As a result, more children, adolescents, and adults with autism are presenting to healthcare providers in various medical specialties for non-psychiatric concerns and various medical comorbidities.

It is well-established that medical comorbidities are more prevalent in children with autism compared to the general population, yet far less is understood about the health status and specific medical conditions affecting adults with autism [3]. Limited studies on adults with autism suggest an increased prevalence of medical comorbidities, mirroring those observed in younger populations [3,4,5]. Recent research further emphasizes the elevated risk of co-occurring physical health conditions and the complex healthcare needs faced by individuals with autism throughout their lives [3]. One study showed that adults with autism experience significantly higher rates of nearly all medical conditions—including immune, gastrointestinal (GI), and sleep disorders, as well as seizures, obesity, dyslipidemia, hypertension, and diabetes—compared to adult controls matched on sex and age [5].

Identifying co-occurring health conditions in individuals with autism can be particularly challenging due to core social and communication deficits that impair clinicians from obtaining accurate medical histories [1,6]. These impairments vary widely in severity, from mild challenges requiring minimal support to profound deficits in communication, cognition, and adaptive functioning [7]. Individuals with the most significant impairments—particularly those with co-occurring ID and minimal or no language abilities beyond age eight—meet criteria for profound autism [8,9]. Introduced by the *Lancet* Commission on the future of care and clinical research in autism, this term distinguishes individuals with high support needs from those with more sophisticated verbal and intellectual abilities [10]. Individuals with profound autism often require intensive, lifelong care and continuous supervision due to substantial functional impairments and complex behavioral needs. While the term “profound autism” has been met with some controversy—particularly among those with autism that do not have co-occurring ID or language impairments who view it as potentially stigmatizing—it serves to highlight a distinct population whose support needs are both extensive and specific. Continued awareness and targeted research are essential to ensure that their unique challenges are recognized and addressed appropriately [8,9,11].

To better characterize the medical vulnerabilities and acute care needs of this population, we conducted a narrative review of the literature using PubMed, PsycINFO, and Google Scholar (January 2000–April 2025). Search terms included “profound autism,” “acute medical events,” “adults with autism,” “intellectual disability,” and “medical comorbidities.” We included English-language, peer-reviewed studies focusing on adults (18+) with ASD and co-occurring ID or limited language, particularly those addressing acute medical presentations and diagnostic challenges. Studies exclusively involving children, non-medical interventions, or lacking empirical data were excluded. We also reviewed reference lists of relevant articles to identify additional sources.

Despite growing awareness of the challenges faced by individuals with profound autism—such as severe communication barriers, sensory sensitivities, and dependence on caregivers for basic health needs—there remains a striking gap in research focused specifically on their non-psychiatric medical care, particularly in adulthood [10,12,13,14]. Acute medical events (AMEs)—defined as sudden-onset health conditions that require timely intervention to prevent serious harm—can be especially difficult to recognize and manage in this population. The unfamiliar equipment and fast pace of clinics and emergency departments (EDs) can be overwhelming and lead to an exacerbation of sensory sensitivities and discomfort. When surrounded by anxiety-provoking stimuli—such as exam table paper, wall-mounted instruments, bright lights, and wristbands—the communication gap widens between patients with profound autism and the healthcare providers trying to assist them [15,16,17]. This challenging environment complicates accurate diagnosis and effective treatment planning, frequently resulting in delays in managing urgent medical comorbidities.

Recent research highlights several key findings related to AMEs and health outcomes in adults with profound autism. First, this population is at heightened risk for hospitalization given their more severe autism symptoms, sleep disturbances, and co-occurring psychiatric conditions [18,19]. Lower adaptive functioning and the presence and severity of ID are strongly associated with concerning behaviors, including self-injurious behavior (SIB) [17,20]. Machine learning models are currently being explored as tools to help clinicians tailor assessment and treatment strategies based on the specific type of SIB a patient exhibits; however, this research remains in its early stages [21]. Providers unfamiliar with the expressive limitations of profound ASD may lead to clinical oversight that manifests as mislabeling nonverbal signs of distress as baseline behaviors or psychiatric symptoms [22,23,24]. Second, individuals with autism and ID often present to EDs not only for medical issues but also for acute behavioral crises, including aggression, suicidal ideation, and SIB. However, systemic barriers—such as limited access to inpatient psychiatric care—frequently impede appropriate intervention [25].

Many acute behavioral exacerbations in individuals with profound autism are driven by underlying medical conditions that often go unrecognized due to communication barriers and diagnostic overshadowing. A multicenter study of 63 adults with autism and ID reported a multimorbidity rate of 84.1%, with prevalence increasing with age. The most common pattern included immune dysfunction, GI disorders, neurological conditions, and joint disease. Medical issues such as epilepsy or GI pain may present as sudden behavioral outbursts or aggression, which are frequently misattributed to primary psychiatric causes. However, when these underlying conditions are accurately identified and treated, marked improvements in behavior and functioning have been observed [19].

These medical vulnerabilities contribute to poor overall health outcomes and reduced life expectancy among individuals with profound autism [14]. Despite the high prevalence of multimorbidity, caregiver reports consistently highlight substantial gaps in healthcare access—particularly during AMEs, when timely recognition and a coordinated, multidisciplinary response are essential. A recent mixed-methods study involving caregivers of adolescents and adults with profound autism identified the most frequently unmet needs as autism-informed primary care, behavioral support services, and crisis response infrastructure. Barriers to accessing these services were strongly associated with socioeconomic disadvantages, minoritized backgrounds, and complex behavioral profiles. Caregivers also emphasized the lack of individualized, goal-oriented services and the need for centralized, coordinated treatment models that extend into adulthood. These findings underscore the urgent need to realign healthcare systems to better address the intersecting medical and behavioral challenges faced by this high-risk population—particularly in the context of acute medical crises [14].

Existing tools such as the Autism Healthcare Accommodations Tool (AHAT) and the Academic Autism Spectrum Partnership in Research and Education (AASPIRE) Healthcare Toolkit represent promising efforts to improve care for autistic adults by facilitating individualized accommodations and improving provider self-efficacy [26,27]. The AHAT is a patient-centered tool designed to help autistic individuals communicate their healthcare needs, sensory preferences, and communication styles to providers. The AASPIRE Healthcare Toolkit includes resources for both patients and providers, including checklists, worksheets, and training materials aimed at reducing barriers to care. These tools have shown promise in enhancing provider confidence and tailoring care to patient needs. However, despite these advances, there remains a critical gap in protocols specifically designed to support the recognition and management of acute medical events in adults with profound autism—particularly those who are minimally verbal and unable to self-advocate in traditional clinical settings.

### 1.2. Common AMEs in the Context of ASD

Acute medical events can range from infections and injuries to life-threatening conditions such as appendicitis, one of the most common examples that present to the ED. With an incidence of approximately 233 cases per 100,000 individuals, appendicitis remains the leading cause of abdominal surgical emergencies worldwide [28]. If not diagnosed and treated promptly, appendicitis can progress to appendix perforation, leading to peritonitis and potentially life-threatening infection [29]. Classic signs of appendicitis typically begin with vague pain around the umbilicus, which later localizes to the right lower quadrant as inflammation progresses. This pain intensifies over several hours and is often accompanied by tenderness, fever, and nausea [30]. However, these hallmark symptoms may be challenging to detect in minimally verbal or cognitively impaired individuals with profound ASD. Due to sensory processing differences and communication barriers, they may struggle to express their pain accurately or at all [31]. This can delay diagnosis and intervention, potentially worsening outcomes due to a lack of clear symptom reporting.

Another common AME that is characterized by significant pain and discomfort is nephrolithiasis (kidney stones). More than half a million people visit an ED annually for this condition, and an estimated one in 10 people will develop kidney stones at some point in their lives [32]. Hypertension, diabetes, and obesity—conditions commonly seen in adults with autism—are all risk factors for developing nephrolithiasis [33,34]. Diagnostic procedures for this condition involve imaging techniques such as abdominal X-rays, computerized tomography (CT) scans, or ultrasounds (U/S) [34]. However, patients with autism may find these procedures challenging due to heightened sensory sensitivities and anxiety, complicating timely diagnosis and treatment [35].

Gastrointestinal disorders, including gastroesophageal reflux disease (GERD), constipation, diarrhea, food allergies, colitis, ulcers, inflammatory bowel disease (IBD), and eosinophilic esophagitis (EoE), are common GI comorbidities in individuals with autism [36]. Eosinophilic esophagitis is an allergic inflammation of the esophagus that involves the accumulation of eosinophils in the esophageal lining. It is an idiopathic condition due to an interaction of genetic, environmental, and immune factors that can be challenging to diagnose [37,38]. Although not as prevalent or widely known as other GI disorders, EoE can lead to significant discomfort and complications for those affected, often requiring specialized management and treatment to address its unique inflammatory response to food allergens and environmental triggers [37]. Commonly reported symptoms of this condition are dysphagia with solid foods, food impaction, pain, reflux, or chest discomfort that may eventually present with emergent acute symptoms due to stricture, dilation, or esophageal rupture [38].

This report reviews the literature on AMEs in adults with profound autism and includes detailed illustrative case descriptions as well as a summary table (Table 1) of three adults with profound autism who presented to healthcare professionals with AMEs—specifically, appendicitis, nephrolithiasis, and EoE. As stated previously, these conditions lead to considerable pain and discomfort that progressively worsens the longer they go untreated and have the potential to turn fatal without prompt diagnosis and intervention. The barriers to effective communication between healthcare providers and patients with profound autism in high-pressure medical settings are underscored.

## 2. Illustrative Cases

### 2.1. Case 1

Mr. A is a 31-year-old Caucasian male who meets DSM-5 criteria for ASD, accompanied by severe ID as defined by the DSM-5, language impairment, generalized anxiety disorder (GAD), and catatonia. He was the product of a normal pregnancy and delivery.

As a baby, he babbled and cooed normally but did not speak his first words until between the ages of three and four years. During his early developmental years, he exhibited signs consistent with autism, including repetitive hand flapping, insistence on sameness, and gaze aversion. Although he preferred to be around others, he also enjoyed spending time alone. As a child, he frequently lined up objects and engaged in limited pretend play. He also demonstrated noise hypersensitivity, which persists to this day.

Mr. A attended a special education school until age 22 years, where he had an Individualized Education Program (IEP). He currently lives with his parents and attends a day program. His medical history includes environmental allergies, eczema, diarrhea, and a seizure disorder. Family history is notable for rheumatoid arthritis in his paternal grandfather and Crohn’s disease in his paternal grandmother.

#### 2.1.1. Acute Medical Event

Mr. A had been medically stable until he began experiencing increased urinary frequency, attempting to use the bathroom every 20–30 min. Although he appeared to have a constant urge to urinate, he did not void with each attempt. His parents initially believed the symptoms might be related to “a virus,” as Mr. A had gone on a field trip with his day program the previous day. However, the urinary frequency persisted through the night, and Mr. A became extremely lethargic. He also vomited—an unusual occurrence for him. Over the next 24 h, Mr. A ate and drank fluids intermittently, though less than usual, and the vomiting continued.

Mr. A’s mother pressed on his abdomen to check for pain; it did not appear distended, and he showed no reaction. The following day, his heart rate exceeded 120 beats per minute, and he exhibited whole-body shivering despite a normal temperature. The next morning, his urine appeared “very dark orange,” prompting his parents to take him to a local urgent care center. Based on the history, the initial differential diagnosis included appendicitis, bowel obstruction, intestinal volvulus, pyelonephritis, and kidney stones. On physical examination, Mr. A flinched and withdrew when pressure was applied to the lower right quadrant of his abdomen. The urgent care physician instructed the parents to take Mr. A to the local ED to rule out acute appendicitis.

At the ED, Mr. A remained tachycardic, and his temperature exceeded 101 °F. Over the next 3–4 h, his mother observed that he appeared pale and increasingly lethargic, lying on the hospital bed with minimal movement. A CT scan of the abdomen was ordered, which revealed a possible perforated appendix.

#### 2.1.2. CT Scan Results

“There is a large amount of inflammatory change in the right lower quadrant with a dilated thickened appendix suspicious for perforated appendicitis with multiple foci of free air noted. No definite drainable fluid collection was present. A large amount of phlegmon is noted surrounding the distal appendix. Numerous reactive mesenteric lymph nodes are present. Wall thickening of the distal small bowel is likely reactive. Surgical consultation is recommended.”

#### 2.1.3. Laboratory Studies

A Complete Blood Count (CBC) with differential, Comprehensive Metabolic Panel (CMP), and urinalysis were obtained. Abnormal values included CBC with differential: white blood cell count = 22.2 × 10^3^/uL (4.0–11.0 × 10^3^/uL), absolute neutrophil count 18.9 × 10^3^/uL (2.2–4.9 × 10^3^/uL), absolute lymphocyte count 0.8x10^3^/uL (1.3–2.9 × 10^3^/uL), absolute monocyte count 2.3 × 10^3^/uL (0.3–0.8 × 10^3^/uL), immature granulocyte count 0.16 × 10^3^/uL (0.00–0.08 × 10^3^/uL); CMP: bilirubin (direct) 0.5 mg/dL (0.0–0.3 mg/dL); Urinalysis: “small” amount of bilirubin, blood 7/HPF (0–3/HPF), color “dark yellow,” protein 30 mg/dL (<30 mg/dL), urobilinogen 4.0 EhrlichU/dL (0.2–1.0 EhrlichU/dL), RBC “present,” WBC “present,” squamous cells “present.”

Surgical removal of the appendix was recommended by the attending surgeon. The appendectomy was completed after a 1.5 h procedure. Due to the location of the perforation, a “small nub” of the appendix was left in place. Mr. A remained hospitalized for six days postoperatively, during which he received intravenous antibiotics. He was discharged home with a prescribed course of oral antibiotics and has done well since.

### 2.2. Case 2

Mr. B is a 36-year-old Caucasian male who meets DSM-5 criteria for ASD with accompanying severe ID as defined by the DSM-5, language impairment, and GAD. He was the product of a normal pregnancy and delivery.

He babbled and cooed normally during infancy. He spoke his first word around 15 months of age and began using 2–3 word phrases by 18 months. His current language consists of phrase speech, much of which is repetitive, including both immediate and delayed echolalia.

During development, Mr. B exhibited signs consistent with autism, such as repetitive finger flicking and gaze aversion. He preferred to be alone rather than with others. He showed a fascination with watching objects spin and engaged in repetitive spinning and lining up of objects. He extensively ordered and arranged items throughout the house and did not demonstrate make-believe play. He also had noise hypersensitivity, which persists to this day.

Mr. B attended a specialized school for students with autism, with an IEP, until age 22 years. He currently lives with his parents and attends a day program.

His medical history is notable for the onset of cluster headaches at age 30 years. During these headaches, he would throw himself on the floor, kick and scream, cover his head with his arms, and squeeze his eyes shut. Prior to these episodes, he showed increased perseverative language. He was initially treated with valproic acid at a therapeutic dosage for two years, which was ineffective. Valproic acid was discontinued, and topiramate was started, which has since been highly effective in reducing the frequency and intensity of his headaches. Additionally, Mr. B has GERD and constipation. Family history includes migraine headaches in his mother and maternal grandmother, cluster headaches in a maternal uncle, and kidney stones in his paternal grandfather and paternal uncle.

#### 2.2.1. Acute Medical Event

Mr. B had been in his usual state of health until one day, during a trip away from home, his father noticed a change in his mood—he was disagreeable, appeared irritable, and refused to eat his breakfast. His father gave him ibuprofen, and shortly thereafter, Mr. B’s typical behavior returned. Later that day, Mr. B again appeared irritable and began pointing at his abdomen and grinding his teeth. Given his history of GERD and constipation, his father wondered whether the change in behavior was due to GI distress or the onset of a cluster headache. There was no blood observed in Mr. B’s urine.

Throughout the day, episodes of apparent significant physical distress and discomfort—lasting between 10 and 30 min—alternated with periods during which Mr. B appeared happy and more like his typical self. However, by that evening, he was pointing to various parts of his body, including his back, buttocks, and abdomen, and was writhing in apparent pain on the sofa. Shortly thereafter, he vomited. He then appeared well again, which led his father to believe that the atypical behaviors were related to GERD.

The following morning, his father called Mr. B’s primary care physician (PCP) and described the signs and behaviors Mr. B had exhibited. The PCP, who had extensive experience caring for individuals with autism, referred Mr. B directly to a urologist, who evaluated him three hours later. By that time, the intermittent episodes of atypical behavior alternating with baseline behavior had resolved. The urologist deduced that Mr. B had likely passed a kidney stone. He reassured the father and Mr. B that the painful episodes were likely over. Although Mr. B had a family history of kidney stones, the urologist hypothesized that the stones were probably caused by topiramate—a known potential side effect of the medication, which had been prescribed for cluster headaches.

#### 2.2.2. Laboratory Studies

A CBC with differential, CMP, lipase, and urinalysis were obtained. Abnormal values included CBC with differential: white blood cell count 13.5 × 10^3^/uL (4.0–11.0 × 10^3^/uL), absolute neutrophil count 6.35 × 10^3^/uL (1.6–6.1 × 10^3^/uL), absolute monocyte count 1.21 × 10^3^/uL (0.2–0.8 × 10^3^/uL); CMP: Blood Urea Nitrogen 21 mg/dL (6–20 mg/dL), creatinine 1.4 mg/dL (0.5–1.2 mg/dL), and “many” bacteria on urinalysis. Ultrasound of the abdomen identified multiple kidney stones bilaterally.

Topiramate was subsequently discontinued, and Mr. B’s cluster headaches have since been treated with pregabalin, with moderate success. Mr. B now has annual follow-up visits with the urologist and undergoes routine abdominal U/Ss. He has remained asymptomatic, and no obstructive kidney stones have been detected on follow-up imaging.

### 2.3. Case 3

Mr. C is a 34-year-old Caucasian male who meets DSM-5 criteria for ASD with accompanying severe ID as defined by the DSM-5, language impairment, and GAD. He was the product of a normal pregnancy and delivery.

During infancy, Mr. C babbled and cooed normally. He spoke his first word at 12 months but never developed phrase speech. By 16 months, he had lost all words and is currently non-speaking.

Developmentally, Mr. C exhibited various forms of self-stimulation, such as placing his hand on his mouth to feel vibrations he produced. He engaged in body rocking but did not toe-walk. Unlike some individuals with autism, he showed no fascination with watching or spinning objects and did not demonstrate imaginative play. Mr. C does not have noise hypersensitivity but is averse to bright lights, often moving away and covering his eyes. When feeling well, he enjoys the company of others but does not engage in typical reciprocal social interactions.

He attended specialized education programs for individuals with autism until age 18 years, with an Individualized Education Program (IEP). He currently lives with his parents.

His medical history includes multiple medication allergies, many causing hives. He had frequent ear infections in childhood and experienced severe eczema. Mr. C had his first grand mal seizure at age 18 years, coinciding with the onset of significant GI issues, including motility problems, digestive pain, and constipation. Family history includes multiple sclerosis in a paternal uncle, alcohol use disorder with intense mood swings in his paternal grandfather, and amyotrophic lateral sclerosis in a distant paternal relative.

#### Acute Medical Event

Since the age of 18 years, Mr. C has experienced intermittent episodes of apparent significant pain and discomfort, manifested as atypical behaviors such as withdrawing into his room and fully shutting down, running up and down the hallway screaming, engaging in SIBs, and pinching his mother. According to Mr. C’s mother, it seemed as though he were “trying to get out of his body” because he appeared to be in so much pain and discomfort. He began demonstrating probable signs of pain after eating or taking his medications. His face would become completely red, prompting his parents to question the etiology of the behavioral changes.

In his early 20s, Mr. C was evaluated by a gastroenterologist. Abdominal laparoscopy revealed that his appendix was twisted and adhered to the inner intestinal wall. An appendectomy was performed. However, despite the procedure, Mr. C’s pain and associated behaviors persisted. He subsequently received treatment for gastritis and gastric ulcers, but the pain continued. Due to the intensity of his discomfort, Mr. C was unable to participate in his structured daily activities.

An upper intestinal endoscopy was performed, revealing esophageal exudates, furrows, edema, rings, and stenosis. Biopsy of the esophagus showed more than 15 eosinophils per high-power field, leading to a diagnosis of EoE. Treatment with steroids was initiated soon thereafter and resulted in near-immediate relief of his pain, discomfort, and related atypical behaviors. Overall, Mr. C has remained healthy since that time.

## 3. Discussion

This review highlights the underrecognized challenges of diagnosing and managing AMEs in adults with profound autism, a population often marginalized in both clinical practice and the medical literature. Through the illustrative cases of Mr. A, Mr. B, and Mr. C—summarized in Table 1—we underscore how profound cognitive and communicative impairments can obscure the presentation of urgent medical conditions, leading to delays in recognition, diagnosis, and treatment. These cases are not simply individual anecdotes; rather, they exemplify a broader pattern seen across the care of adults with profound autism—one in which behavioral and communication barriers are misinterpreted or dismissed, often with serious or life-threatening consequences.

Mr. A’s case demonstrates how acute appendicitis can go unrecognized when pain is expressed not through language but through behavioral shifts—withdrawal, lethargy, and vomiting. In a neurotypical adult, such symptoms would likely trigger prompt investigation. But in Mr. A’s case, these signs were initially attributed to a benign viral illness, a delay that permitted progression to perforation and potential sepsis. This clinical oversight reflects a recurring theme: providers unfamiliar with the expressive limitations of profound ASD may mislabel nonverbal signs of distress as baseline behaviors or psychiatric symptoms [12,18]. Recognizing atypical, often subtle indicators of medical distress is not an ancillary skill—it is essential to safe and equitable care for this population.

Mr. B’s experience further reinforces this theme. His behavioral changes—irritability, abdominal pointing, and episodic discomfort—were initially misattributed to known conditions like GERD or assumed to be behavioral manifestations of his ASD. Only after escalation did his father consult a physician who, with specialized knowledge, identified a passed kidney stone as the likely cause. Nephrolithiasis, when not promptly diagnosed, poses risks including infection, renal injury, and even sepsis. Mr. B’s ability to return to baseline between painful episodes also likely contributed to a false sense of reassurance. These complexities underscore the need for clinicians to avoid diagnostic overshadowing—a cognitive bias in which all new symptoms are attributed to an existing diagnosis, in this case, ASD.

Mr. C’s story, while less acute, provides an important counterpoint. His years-long struggle with undiagnosed EoE highlights how chronic conditions may also present as behavioral disturbances in adults with profound ASD. Despite numerous evaluations, his pain was interpreted through a psychiatric lens, delaying an eventual diagnosis that was revealed only after significant deterioration in quality of life. Eosinophilic esophagitis, if untreated, can result in strictures, dysphagia, and malnutrition. His case illustrates the insidious consequences of systemic clinical neglect—where the absence of communicative clarity leads to years of unnecessary suffering.

## 4. Conclusions

Collectively, these cases underscore the critical need for medical professionals to develop a more nuanced and informed approach to adults with profound autism, especially those with limited language abilities. Relying on ASD as a default explanation for diverse symptoms risks obscuring underlying medical conditions that require urgent attention. This interpretive bias—attributing behavioral changes primarily to autism—represents a path of least resistance that compromises comprehensive assessment and clinical rigor. As demonstrated by Mr. A, Mr. B, and Mr. C, such oversimplification can lead to missed diagnoses, delayed treatment, and potentially life-threatening outcomes.

## 5. Future Directions

Addressing the diagnostic and care disparities faced by adults with profound autism requires a deliberate, multi-level response. Medical education must be expanded to include comprehensive training on developmental disabilities, with specific attention to recognizing atypical presentations of both acute and chronic medical conditions. Providers must be trained to identify nonverbal or behavioral cues as legitimate signs of medical distress rather than attributing them solely to psychiatric or developmental diagnoses.

In addition, clinical systems should foster collaborative models of care that integrate caregiver observations into medical evaluations [39]. Caregivers are often the most reliable historians for individuals with profound autism and can detect subtle deviations from baseline functioning. Their insights must be treated as clinically valuable data points, not anecdotal or secondary information.

To reduce the risk of delayed diagnoses during AMEs parents and practitioners should proactively develop individualized health profiles that document baseline behaviors, typical pain responses, and known comorbidities. These tools—such as behavior tracking logs or communication passports—can serve as critical references during medical crises. Establishing relationships with autism-informed primary care providers and ensuring routine health screenings (e.g., GI evaluations, neurological assessments) can help identify medical vulnerabilities before they escalate. Practitioners should also work with families to create care plans that outline preferred communication methods, known triggers, and behavioral indicators of pain or distress, thereby improving recognition and response during acute episodes.

Finally, healthcare systems must develop and implement standardized protocols for evaluating acute behavioral changes in patients with limited communication. These protocols should prompt clinicians to consider—and rule out—underlying medical causes early in the diagnostic process. Improving diagnostic accuracy and timely intervention in adults with profound autism will not only prevent unnecessary suffering but also reflect a broader commitment to health equity for one of medicine’s most vulnerable and often invisible populations.

## Figures and Tables

**Table 1:** Summary of three patients across core clinical domains, including age, co-occurring conditions, behavioral presentation, communication abilities, diagnostic delays, and outcomes.

	Mr. A	Mr. B	Mr. C
Age (years)	31	36	34
Core Diagnoses	ASD, ID, language impairment, GAD, catatonia	ASD, ID, language impairment, GAD	ASD, ID, language impairment, GAD
Language Ability	Limited verbal communication	Phrase speech with echolalia	Non-speaking
Baseline Behavior	Friendly, prefers routines, socially interested	Socially avoidant, intense ordering behavior	Enjoys company, avoids bright lights
Behavioral Signs of AME	Lethargy, vomiting, increased urination, shivering	Irritability, pointing, writhing, vomiting	Self-injury, withdrawal, screaming
Medical Condition	Perforated appendicitis	Nephrolithiasis (likely topiramate-induced)	Eosinophilic esophagitis (EoE)
Diagnostic Delay Factors	Atypical symptom presentation; communication barriers	Symptoms mistaken for GERD or headache	Symptoms attributed to psychiatric causes
Intervention	Appendectomy with IV and oral antibiotics	Medication change (discontinued topiramate)	Endoscopy, steroid treatment
Clinical Outcome	Recovered well post-surgery	Headaches managed with pregabalin; asymptomatic	Symptoms resolved with treatment

## Data Availability

The original contributions presented in the study are included in the article, further inquiries can be directed to the corresponding author.

## Abbreviations

The following abbreviations are used in this manuscript:

ASD	Autism spectrum disorder
AME	Acute medical event
ID	Intellectual disability
GI	Gastrointestinal
EoE	Eosinophilic esophagitis
